# Successful surgical aortic valve replacement in a patient with progeria

**DOI:** 10.1093/icvts/ivac115

**Published:** 2022-05-06

**Authors:** Petar Vukovic, Petar Milacic, Igor Zivkovic, Dragana Kosevic, Slobodan Micovic

**Affiliations:** Department of Cardiac Surgery, Dedinje Cardiovascular Institute, Belgrade, Serbia

**Keywords:** Progeria, Aortic valve stenosis, Aortic valve replacement

## Abstract

The progeroid syndrome includes a group of rare, severe genetic disorders clinically characterized by premature physical ageing. Severe aortic stenosis has been described in progeria patients, but no previous surgical aortic valve replacement was reported. We describe a successful surgical aortic valve replacement combined with coronary artery bypass grafting in a progeria patient with severe aortic stenosis and a small aortic annulus.

## INTRODUCTION

Progeroid syndrome (PS) includes a group of rare, severe genetic disorders clinically characterized by premature physical ageing [1]. The life expectancy is poor due to the early development of severe cardiovascular pathology. Severe aortic stenosis has been described in PS patients, but surgical aortic valve replacement has not been previously reported [2]. We describe a successful surgical aortic valve replacement combined with coronary artery bypass grafting in a progeria patient with severe aortic stenosis and a small aortic annulus.

## CASE REPORT

**Figure 1:**
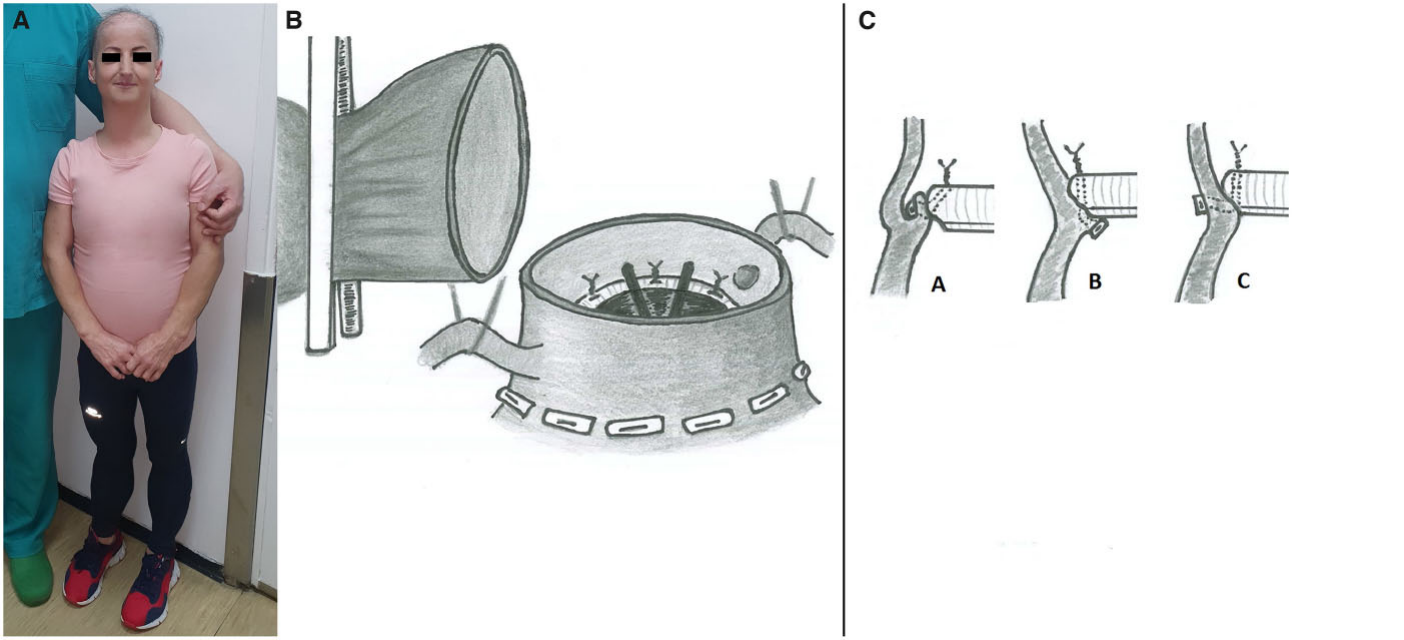
(**A**) The distinctive physical appearance of the patient with progeroid syndrome. (**B**) The ascending aorta is completely divided above the sinotubular ridge. The coronary arteries are mobilized and secured with rubber tapes. (**C**) The pledgeted horizontal mattress sutures are placed above the aortic annulus. The ventricular placement of the pledgets allows the supra-annular position of the prosthesis. Transaortic placement of the sutures eliminates the presence of pledgets in the left ventricular outflow tract.

A 33-year-old woman who suffered from chest pain, dyspnoea and fatigue due to severe aortic stenosis was admitted for aortic valve intervention. The patient characteristics are presented in Table 1 and Fig. 1A. The diagnosis of PS was confirmed by genetic testing, which revealed non-classic progeria-intron A mutation c. IVS11 + 5G>A (c.1968 + 5g>A).

**Table 1:** Preoperative, intraoperative and postoperative patient characteristics

Patient characteristics	− 33-year old
	− 143 cm height, 40 kg weight
	− Body mass index 19.8
	− Body surface area 1.26 m^2^
	− Gout
	− Diabetes mellitus
	− No cerebrovascular events
Patient physical appearance	Short stature, alopecia, prominent eyes, loss of eyelashes and eyebrows, a ‘beaked’ nose, scleroderma-like skin changes and micrognathia.
	She had prominent joints, loss of subcutaneous tissue and dystrophic nails. All cognitive and intellectual functions were well developed.
Preoperative echocardiography parameters	− Aortic valve area 0.3 cm^2^
	− Peak/mean gradient 130/81 mmHg
	− Vmax 5.7 m/s
	− Aortic valve regurgitation 2+
	− Aortic annulus diameter 18 mm
	− Ascending aorta diameter 28 mm
	− EF 40%
	− MR 1+
Coronary angiography	− LAD stenosis 70%
Operative data	− St Jude regent No 17 mm mechanical valve
	− Predictive IEOA 1.48 cm^2^/m^2^
	− Internal thoracic artery to LAD bypass
Postoperative echocardiography parameters	− Peak/mean gradient 30/18 mmHg
	− Vmax 2.7 m/s
	− EOA 1.2 cm^2^
	− iEOA 0.95 cm^2^/m^2^
Follow-up	− 13 months
	− Peak/mean gradient 32/19 mmHg
	− Uneventful

EF: ejection fraction; EOA: effective orifice area; iEOA: indexed effective orifice area; LAD: left anterior descending artery; MR: mitral regurgitation.

Echocardiographic parameters are presented in Table 1. The patient was thoroughly informed about the treatment options, including percutaneous and surgical approaches for aortic valve replacement. The advantages and disadvantages of each approach were explained. The patient decided to be treated with the most durable option, thus insisting on surgical aortic valve replacement with mechanical prosthesis and left anterior descending artery bypass grafting with the left internal mammary artery.

The operation was performed through the median sternotomy and central arterial and venous cannulation. A transversal aortotomy revealed senile degeneration of the aortic leaflets, which were meticulously removed. There was no annulus calcification or subvalvular membrane. In order to implant the mechanical prosthesis size 17 (ST Jude Regent) in a very small aortic annulus, the ascending aorta was completely transected above the sinotubular ridge. In addition, the coronary arteries were carefully mobilized and secured with rubber tapes (Fig. 1B). This approach enabled the placement of all interrupted pladgeted sutures from the outside of the aortic root towards the inside of the aortic annulus (transaortic sutures; Fig. 1C). The prosthesis was implanted in the supra-annular position. Left internal mammary artery graft was anastomosed to the proximal left anterior descending artery. The postoperative period was uneventful.

## DISCUSSION

The estimated prevalence of patients with PS is one in 4–8 million new births. *Progressive atherosclerosis* is a significant pathological finding which leads to early death caused by myocardial infarction, heart failure and stroke. In the classical Hutchinson–Gilford progeria syndrome, most common patients die from myocardial infarction at 13 years of life. The adult form of progeria (Werner syndrome) has a longer life expectancy, and most often, patients die of myocardial infarction in the mid-50s [3].

Aortic valve implantation in a small aortic annulus may be technically challenging. The ideal solution for treatment does not exist. Several root enlargement techniques were introduced to increase the size of implanted prostheses and avoid the patient-prothesis mismatch. The surgeon must weigh up the benefit of root enlargement against the risk of intraoperative or postoperative adverse events and mortality [4]. The simple interrupted suture technique demonstrated a larger prosthesis–annulus size ratio. The concern about this technique is due to the increased risk of paravalvular leak [5]. In our case, the intraoperative sizing showed that the 17 mm mechanical prosthesis may barely fit into the aortic annulus. Considering the patient’s body surface area of 1.26 m^2^ and projected indexed effective orifice area, there was no danger of patient-prosthesis mismatch with 17 mm prosthesis size. It was previously demonstrated that prosthesis implantation using interrupted mattress sutures with the pledgets placed on the left ventricular side of the annulus (the supra-annular implantation technique) allowed the use of a larger prosthesis for a given annulus than the intra-annular implantation technique with the pledgets located above the aortic annulus. However, the pledgets placed on the ventricular side of the annulus may obstruct the left ventricular outflow tract and decrease the effective orifice area of the prosthesis (Fig. 1C). This is particularly the case in a patient with a very small aortic annulus [6]. Therefore, we decided to use transaortic sutures and the above-described implantation technique to implant the prosthesis in the supra-annular position. We found that this approach may be suitable for patients with small aortic annuli, complex cases of infective endocarditis and redo surgery.

## CONCLUSION

The annular diameter and body surface area ratio in the patients with PS decrease the possibility of patient prosthesis mismatch (PPM). Transaortic or simple interrupted sutures placement technique could be a satisfactory alternative to the aortic root enlargement technique, which requires an advanced surgical skill level.

**Conflict of interest:** none declared. 

**Reviewer information:** Interactive CardioVascular and Thoracic Surgery thanks Jose G. Fragata and the other anonymous reviewer(s) for their contribution to the peer review process of this article.
